# Challenging the Surface Area Paradigm: A Critical Review of Low-BET Metal–Organic Frameworks for the Adsorption of Organic Water Pollutants

**DOI:** 10.3390/nano16191271

**Published:** 2026-10-08

**Authors:** Malcom Frimpong Dapaah, Xingbao Zhu, Hui Jia, Huiying Zhang, Ruth Antwi-Baah, Candy Ann Dwamena Darkwa, Liang Cheng

**Affiliations:** 1 School of Agricultural Engineering, Jiangsu University, Zhenjiang 212013, China; mfdapaah@ujs.edu.cn; 2 Technical Centre for Soil, Agriculture and Rural Ecology and Environment, Ministry of Ecology and Environment, Beijing 100012, China; 3 School of the Environment and Safety Engineering, Institute of Environment and Ecology, Jiangsu University, Zhenjiang 212013, China; 1000005403@ujs.edu.cn; 4 School of Environment, Tsinghua University, Beijing 100084, China; ying_zz1995@163.com; 5 College of Ecology and Environment, Chengdu University of Technology, Chengdu 610059, China; maadede97@gmail.com; 6 Department of Materials and Metallurgical Engineering, College of Engineering, Kwame Nkrumah University of Science and Technology, Kumasi AK-448-7038, Ghana; candydarkwa@gmail.com

**Keywords:** metal–organic framework, adsorption, low surface area, Brunauer–Emmett–Teller, water treatment

## Abstract

The application of metal–organic frameworks (MOFs) toward the adsorption of organic pollutants from aqueous systems has attracted heightened attention in recent times. While considerable research works have examined high-surface-area MOFs, growing evidence demonstrates that MOFs with low Brunauer–Emmett–Teller (BET) surface areas (*S*_BET_ ≤ 100 m^2^/g) can also achieve remarkably high pollutant adsorption. This review critically examines, for the first time, recent developments that have enabled low-surface-area MOFs to adsorb organic pollutants from wastewater efficiently. The first part explores compositional modifications of low-surface-area MOF adsorbents, with particular attention to their metal centers. Furthermore, four categories of auxiliary components were delved into. Additionally, the factors influencing adsorbent synthesis were presented. Moreover, low- and high-*S*_BET_ MOFs were directly compared. Finally, current challenges and key research gaps were identified. Future research priorities such as rigorous mechanistic validation, performance evaluation in realistic water matrices, improved regeneration strategies, and advancing pilot-scale studies to facilitate practical application were also presented.

## 1. Introduction

Industrial growth to meet the increasing daily demand has led to over 7 × 10^6^ tons of industrial waste being discharged into water streams each year, introducing organic contaminants into the environment through wastewater [1,2]. These pollutants accumulate in the body and can affect the immune, endocrine, and cardiovascular systems [3]. Given the health impacts and characteristics of these organic contaminants (Table S1), achieving cleaner water is imperative.

Prominent techniques for removing contaminants encompass reverse osmosis, chemical precipitation, catalysis, adsorption and biological approaches [4,5]. The biological routes, such as wetlands, biofilms, microbial genetic manipulation, bioaugmentation, and biosparging, tend to be time-consuming, although environmentally benign with anaerobic or aerobic modifications [6,7,8,9]. The adsorption route, on the other hand, offers high efficiency, reusability, affordability, and ease of operation [10].

Numerous porous materials, such as carbons, silica, resins, zeolites, and metal–organic frameworks (MOFs), have demonstrated significant adsorption capacities. MOFs, which consist of metal nodes connected by organic ligands via coordination bonds, are known for their tunable porosity and large surface areas [11,12,13,14,15,16]. During oxidation, they prevent the formation of clusters, a problem observed in nano-zero-valent iron particles. They possess high densities of catalytic positions at their metal centers relative to zeolites [17,18]. These distinctive properties make MOFs preferable for a wide range of applications, including the purification of polluted air and water, sensors, food packaging, gas storage, pharmaceuticals, catalysis, and adsorption [19,20,21,22,23,24,25].

A careful analysis highlights the substantial amount of research on MOFs as adsorbent materials. Interestingly, there has been a surge in MOFs with lower Brunauer–Emmett–Teller (BET) surface areas. Although several reviews have explored various segments of MOFs related to adsorption in wastewater [24,25,26,27,28], specific focus on MOFs with these low surface areas as adsorbents for organic pollutants has not been addressed.

This review, for the first time, highlights recent strategies involving low-surface-area MOFs towards the adsorption of organic contaminants in wastewater. Most importantly, we evaluated the contributions of metal centers, auxiliary components, and synthesis factors in recent MOF designs. In addition, low- and high BET surface area (*S*_BET_) MOFs were directly compared. Finally, we addressed ongoing challenges and future directions. Rather than covering every study on MOFs as adsorbents, this review focuses on recent pivotal advancements in organic pollutant adsorption. These insights can guide researchers in optimizing MOFs for more effective removal of persistent organic contaminants.

## 2. Literature Retrieval, Classification and Validation Framework

### 2.1. Literature Retrieval and Study Selection

**Figure 1:**
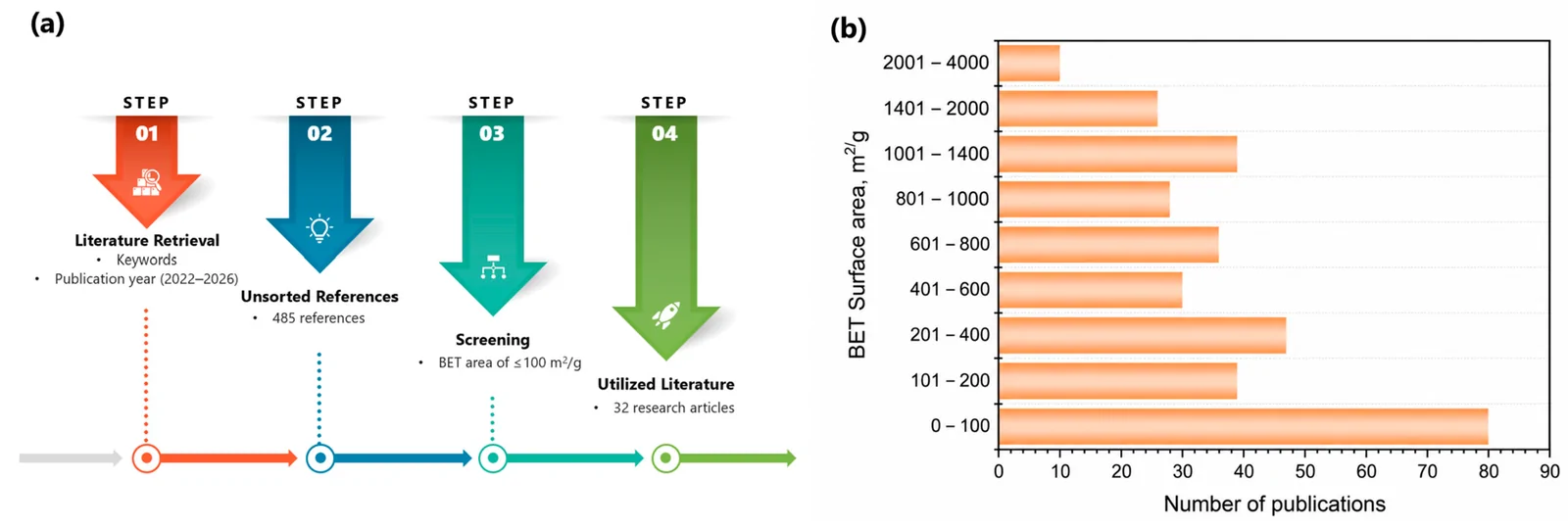
(**a**) Outline of literature retrieval and inclusion criteria, (**b**) reported BET surface areas vs. number of publications.

This study presents a critical narrative review supported by a literature search on the Web of Science database to examine the adsorption performance, structural characteristics, and governing mechanisms of low-BET MOFs for organic pollutant removal. The initial search was performed in August 2025 and subsequently updated in April 2026 to capture recent advancements in the field. The search was restricted to peer-reviewed papers published in English, employing the keywords adsorption, organic pollutant, wastewater treatment, and metal–organic frameworks. This strategy yielded a total of 485 records, predominantly published within the past 4 years. The identified publications were screened based on their titles, abstracts, and full texts, and they were included only if they explicitly addressed the adsorption of organic pollutants in aqueous systems using metal–organic frameworks. A total of 32 research articles that aligned with the thematic focus were selected for comprehensive analysis in the main review. Additional pertinent studies were incorporated selectively to support discussions in other sections where relevant. This methodological approach was designed to provide a focused yet representative overview of the current state of research; however, as a narrative review, it does not constitute a systematic analysis. Hence, the studies here are not a statistically representative sample of all published work on low-BET MOFs. These steps are summarized in Figure 1a.

### 2.2. Operational Definition of Low-BET MOFs

In this review, 100 m^2^/g is used as a predefined operational threshold rather than as an intrinsic property of a material class. Two considerations justify this choice. First, it is data-driven. Of the records retrieved, the 0–100 m^2^/g interval contained the largest number of studies, and its upper boundary coincided with a natural gap in the reported BET distribution (Figure 1b), so that the cutoff separates, rather than arbitrarily splits, the surveyed population. Second, it is benchmark-relative. The benchmark MOFs most frequently applied to aqueous adsorption lie approximately one order of magnitude above the cutoff. For example MIL-101 (>3000 m^2^/g) [26], ZIF-8 (>1500 m^2^/g) [27], MIL-125(Ti) (>1000 m^2^/g) [28], UiO-66 (>1000 m^2^/g) [29] and MOF-5 (3000 m^2^/g) [30], so *S*_BET_ ≤ 100 m^2^/g unambiguously identifies materials that would be described as low-surface-area in the MOF literature. The threshold is therefore a classification device as it defines the inclusion set and carries no performance claim. Materials above this value may exhibit comparable or higher adsorption performance. This classification facilitates comparison of low-BET systems and assessment of the roles of surface chemistry, pore accessibility, and framework structure in adsorption. Two sub-classes followed directly from it and are used in Section 5 (ultra-low-surface-area MOFs; *S*_BET_ < 20 m^2^/g and moderate-low-surface-area MOFs; *S*_BET_ = 20–100 m^2^/g).

Herein, ‘low-surface-area MOFs’ refers to adsorbent systems with a measured S_BET_ of ≤100 m^2^/g. This encompasses both intrinsically low-surface-area MOFs and composite-derived materials, in which conventionally high-surface-area MOFs are combined with secondary matrices that reduce the overall *S*_BET_. Although the origin of the low surface area differs, both categories satisfy the selection criterion and provide valuable insight into adsorption mechanisms governed by intrinsic framework chemistry and by matrix–MOF interfacial synergy.

### 2.3. Verification of Framework Integrity

A low measured BET surface area does not, by itself, establish an intrinsically low-porosity framework. In MOFs expected to be highly porous, unusually low *S*_BET_ values may instead arise from incomplete activation, residual solvent or modulators, framework collapse, or formation of impure or partially amorphous phases. Including such values without structural verification could therefore propagate uncertainties from the primary literature. Accordingly, studies included in the core analysis were re-examined for evidence confirming the intended crystalline and chemically consistent framework, with the available evidence summarized in Table 1. Three criteria were considered: (i) PXRD consistency with the expected topology, based on simulated patterns or reference data; (ii) compositional agreement with the proposed structure, supported by XPS, elemental analysis, ICP-OES, or SEM-EDX; and (iii) a structurally plausible explanation for the low S_BET_. Low-*S*_BET_ materials were further classified as intrinsic or composite-derived. Entries marked ‘not reported’ indicate limitations in the primary studies rather than validation of the reported values. Studies reporting unusually low *S*_BET_ for nominally porous MOFs without adequate crystallographic or compositional evidence were excluded from the core analysis.

### 2.4. Scope, Selection Bias and Limitations

The conclusions of this critical narrative review are subject to some limitations. First, the search was restricted to a single database (Web of Science) and English-language, peer-reviewed articles, potentially excluding studies indexed elsewhere or in the grey literature. Second, the literature is weighted toward recent publications, meaning that some reported findings may not yet have undergone extensive independent reproducibility. Third, publication and selection biases cannot be excluded, particularly because the studies in Section 3, Section 4 and Section 5 were purposively selected for their mechanistic relevance and coverage of major adsorbent and pollutant classes rather than as a complete or random sample. Accordingly, comparisons in Section 5 represent qualitative patterns rather than statistically generalizable trends. Figure 1b indicates research concentration rather than a comprehensive bibliometric census, and the reported frequency of mechanisms should not be interpreted as their prevalence or relative importance across the field. Importantly, potential uncertainty in reported low-BET values arising from incomplete activation or phase impurities was assessed directly for individual materials in Section 2.3.

### 2.5. Critical Implications of the Literature Selection and Validation Framework

The screening and validation of the selected studies highlighted an important issue in interpreting low-BET materials. The reported *S*_BET_ should not be considered independently of framework structure, activation conditions, pore accessibility, and material composition. The reviewed studies include both intrinsically low-surface-area MOFs and composite materials. These situations should be distinguished because a low BET value caused by pore blocking or dilution of the MOF matrix has a different meaning from one resulting from framework flexibility, limited nitrogen accessibility, or inherently low porosity. At present, the literature does not provide a consistent approach for making this distinction. Future studies should therefore provide more detailed information on activation conditions, PXRD patterns, material composition, BET fitting parameters, pore-size distributions, and, where possible, independent characterization of accessible adsorption sites. A more consistent reporting approach would improve the comparisons among low-BET materials and provide stronger evidence for evaluating whether high adsorption performance can occur independently of high surface area.

## 3. Compositional Modifications

### 3.1. Metal Sites

The type of metals utilized for MOF adsorbent synthesis can influence the magnitude of surface area exhibited and, more importantly, the pollutant adsorption capacity. During metal variations, the different coordination of organic ligands to metal sites can lead to the formation of structural isomers. These configurations are referred to as polymorphic MOFs [61]. For instance, the regular octahedral MIL-101(Cr) morphology can be transformed into elongated MIL-53 rods upon Fe addition, resulting in improved pollutant adsorption [22]. Such an occurrence was attributed to the MIL-53 ‘breathing’ property, which triggers expansion or contraction in response to guest pollutant molecules. The reversible nature of this framework was due to the μ–OH bridge, which formed hydrogen bonds with incoming polar contaminants [62]. The hydrated Fe unit cell volume and channel diameters then increased, without bond destruction (from 899.6 Å^3^ and 6.759 Å to 1973.5 Å^3^ and 7.518 Å, respectively) [63,64]. Adsorbents with Fe addition ≥ 50 wt% exhibited type I physisorption (MIL-101) characterized by micropores, whereas those with Fe content < 50 wt% experienced type IV isotherms (MIL-53), denoting mesoporous surfaces (H3 with a hysteresis loop). Therefore, notwithstanding the reduced surface area, which is similar to MIL-53 *S*_BET_ values reported by Abdollahi et al. [65] (14.04 m^2^/g) and Vu et al. [66] (14.5 m^2^/g), mesoporous MIL-53 afforded greater adsorption of p-nitroaniline compared with other large surface area adsorbents. Importantly, MIL-53 MOFs exhibited a flexible framework that can adjust pore dimensions to accommodate guest molecules, resulting in a reversible breathing effect that alters cell volume, while preserving structural integrity [67]. During adsorption, the narrow-pore MIL-53 transforms toward a large-pore configuration, increasing accessible pore volume and exposing previously inaccessible adsorption sites. The reversible nature of this phase transition enables adsorption capacity to be largely retained over repeated adsorption–desorption cycles, provided framework crystallinity is preserved. In detail, hydration increases the separation between adjacent Fe centers, expanding the channel diameter from 6.759 Å to 7.518 Å and the unit cell volume from 899.6 Å^3^ to 1973.5 Å^3^ [62,63]. This hydration-induced structural flexibility enables MIL-53 to open its pore channels only in the presence of guest molecules, thereby enhancing the accessibility of adsorption sites. As a result, MIL-53 can achieve high adsorption performance despite its relatively low *S*_BET_ and pore volume. This played a key role during p-nitroaniline adsorption. Additionally, π–π interactions and hydrogen bonding were identified by XPS and FTIR, respectively.

In addition, bimetallic MOFs can still exhibit promising adsorption properties when grown onto nanofibers to improve reusability. In an affiliated study, Lin et al. [52] adsorbed 164.20 mg/g of naproxen using (PAN=polyacrylonitrile) PAN@UiO-66 (Zr/Hf). Bimetallic UiO-66 (Zr/Hf) was coated onto the PAN fibers via ligand-triggered assembly. Naproxen adsorption was in the order: PAN/2-amino-terephthalic acid (BDC) nanofiber membrane < cross-linked PAN/BDC nanofiber membrane < PAN/UiO-66 (Hf) < PAN/UiO-66 (Zr) < PAN@UiO-66 (Zr/Hf). The enhanced adsorption of PAN@UiO-66 (Zr/Hf) was due to the porous architecture of UiO-66 (Zr/Hf), which substantially increased the number of active positions. The combined adsorption action in the bimetallic UiO-66 (Zr/Hf) mainly stemmed from the dual roles of the metallic centers and defective sites. The defect sites contained reactive Hf-OH/Zr-OH entities [68] that strongly connected with the -COOH groups of naproxen through coordination or hydrogen bonding. Moreover, the electronic correlation between Hf and Zr enhances the coordination strength of the metal nodes and adjusts the electronic distribution within the MOF unit [69]. This amplified π–π interactions between the naphthalene rings in the pollutant and the aromatic rings in the ligands and increased electrostatic interactions. XPS analysis further confirmed metal complexation, hydrogen bonding, π–π and electrostatic interactions.

### 3.2. Auxiliary Components

**Figure 2:**
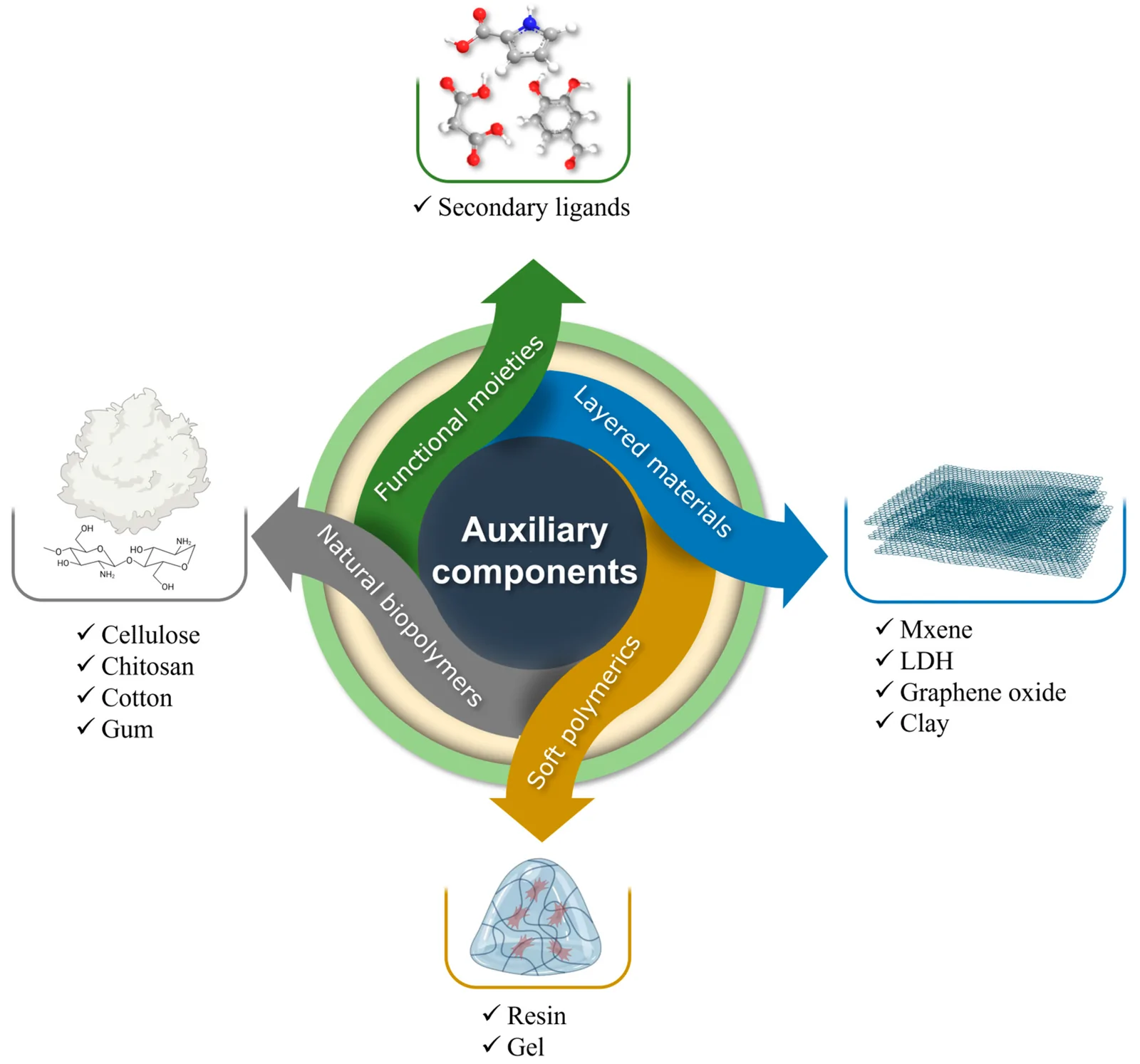
Auxiliary components utilized in MOFs for pollutant adsorption.

The incorporation of various auxiliary components into the ligand or metal sites can influence the adsorption efficiency. This section is therefore classified into functional moieties, layered materials, soft polymerics and natural biopolymers (Figure 2).

#### 3.2.1. Functional Moieties

##### Secondary Ligand

Undoubtedly, organic ligands constitute a crucial part of the MOF structure. The introduction of secondary ligands can influence the pore size of the adsorbent openings. In a related study, five secondary ligands (diethylenetriaminepentaacetic acid (dtpa), 3,4-dihydroxybenzaldehyde (dhba), pyrrole-2-carboxylic acid (pca), cinnamaldehyde (cin), and malonic acid (mal)) were incorporated into the MIL-88A(Fe) structure [23]. Adding 5 wt% of the ligands led to an increase in adsorption capacity towards tetracycline hydrochloride (TCH) following the sequence: -dtpa < -cin < -mal < -pca < -dhba. The top performance of the dhba adsorbent was attributed to its wider pore size of 3.414 nm, as determined from the Barrett-Joyner-Halenda (BJH) pore size distribution. This enabled efficient pore filling with TCH entities (molecular size of 1.5 nm). Electrostatic interactions, π–π interactions from the aromatic rings, and H-bonding via the electronegative O were confirmed by zeta potential, FTIR, and XPS, respectively. Notwithstanding the low surface area (15.48 m^2^/g) of MIL-Fe:In(25:75)-5%-dhba, 97.88% was achieved for TCH removal. The physisorption isotherms aligned with type IV isotherms (H3 group), which indicated the mesoporous nature of the adsorbent [70].

The post-synthetic modification of MOFs with methyl iodide (MeI) and sodium chloride (NaCl) can induce favorable ionic properties in the adsorbent. Owing to this, Mukherjee et al. [47] developed a cationic Hf-based 1′@MeI MOF to remove diclofenac, naproxen, and ibuprofen. It showed high adsorption capacities of 482.9, 295.9, and 219.5 mg/g, respectively. Methylation reduced pore accessibility but created positively charged sites that strongly attracted the negatively charged drugs. XPS, pH studies, and DFT confirmed that electrostatic attraction was the main adsorption mechanism.

#### 3.2.2. Layered Materials

##### Mxene

Aside from metal sites, another approach to improving adsorption in low-surface-area MOFs is to introduce alkalized MXene nanosheets derived from 2D transition-metal nitrides or carbides. From an allied work, Wang et al. [45] demonstrated 98% TC adsorption utilizing Co-MOF interlaminated with alkalized MXene sheets. Higher pore volume was observed in D-MXene/Co-MOF_0.15g Co2+_ = 0.036 cm^3^/g, compared to Co-MOF = 0.013 cm^3^/g and D-MXene = 0.015 cm^3^/g. This enabled better diffusion of the pollutant and provided more active adsorption sites. Also, a significant reusability efficiency of over 95% was achieved with the composite membrane even after 10 adsorption–desorption cycles. In addition to the MOF-induced nanochannels, the other adsorption mechanism of the composite was determined by XPS and FTIR analysis to involve π–π stacking, electrostatic interactions, and hydrogen bonding. Density functional theory (DFT) calculations were conducted to further clarify the adsorption mechanism of Co-MOF towards TC and Cr(VI). This was achieved by evaluating the adsorption configurations and energies of TC and HCrO_4_^−^ onto Co-MOF. The deduced adsorption energies were −3.2 eV for TC and −1.98 eV for HCrO_4_^−^, revealing a stronger interaction between Co-MOF and TC. Both values being below −0.4 eV confirmed that the adsorption phase was chemically induced [71].

##### Layered Double Hydroxides

Layered double hydroxides (LDHs) are a class of two-dimensional inorganic lamellar compounds represented by the formula [M*^II^*_1−x_M*^III^*_x_(OH)_2_]^z+^(A^n−^)z/n•*m*H_2_O, with M*^III^* and M*^II^* denoting trivalent and bivalent cations, respectively [72,73]. The addition of LDH units can effectively suppress the aggregation of MOFs, thereby increasing the exposure of coordinatively unsaturated cationic metal centers and significantly improving the adsorption performance [74]. This is plausible, as they possess excellent ion-exchange capacity, abundant intercalated anions, and positively charged, highly porous nanosheets, making them suitable for promoting pollutant removal [75]. In a related study, Luo et al. [44] prepared MIL-88B(Fe)/ZnTi-LDH, which exhibited higher tetracycline adsorption compared to both MIL-88B(Fe) and ZnTi-LDH (q_eMIL-88B(Fe)/ZnTi-LDH_ = 543.89 mg/g vs. q_eMIL-88B(Fe)_ = 373.37 mg/g and q_eZnTi-LDH_ = 365.25 mg/g). Similarly, the Yilmaz group [43] prepared MIL-88A/NiFe-LDH@SA, which displayed 94.82% adsorption of tartrazine. The adsorption mechanism was inferred to involve Yoshida hydrogen-bonding interactions between the carboxyl and hydroxyl groups of the adsorbent and the benzene rings of tartrazine [76]. The FTIR spectrum after tartrazine adsorption revealed a decrease in the C–O band intensity and a shift in the –OH band, indicating hydrogen-bonding interactions with tartrazine. The peak shift from 1600 to 1594 cm^−1^ implied π–π interactions between the aromatic rings of MIL-88A and tartrazine. They also deduced electrostatic interactions from the point of zero charge (PZC) analysis.

##### Graphene Oxide

Apart from LDH, graphene oxide (GO) also reduces MOF agglomeration and enhances dispersion interactions [77]. GO sheets are decorated with various oxygenated groups, including carbonyl, epoxy, carboxyl, and hydroxyl groups, distributed across the basal surface and layer peripheries, which can enable desired interactions with contaminants [78,79]. Sethi et al. [42] demonstrated 97.7% adsorption of tetracycline using CuBTC-GO (CBG11), whereas pristine CuBTC and GO afforded ~72% and ~65% removal, respectively. This behavior was attributed to the synergistic integration of both components, thereby boosting the abundance of accessible adsorption centers. The adsorption of tetracycline was driven by hydrogen bonding, as evidenced by FTIR analysis. FESEM, elemental mapping and N_2_ sorption curves confirmed the attachment of tetracycline to the composite surface and its penetration into the pores. Also, electrostatic and π–π interactions were inferred.

The conventional physical mixing method for dispersing MOFs across GO sheets can sometimes lead to non-uniformity, especially in vacuum-filtered membranes [80]. Hence, adopting a nacre-inspired ‘brick-and-mortar’ arrangement can promote structural uniformity in MOF/GO adsorbents. From this standpoint, Zhang et al. [41] engineered a composite system in which a spindle-shaped MOF served as the structural backbone, GO as the elastic bridging material, and tannic acid as the cross-linking mediator. The fabricated CoFe-MOF/GO-TA exhibited 98.45% separation efficacy (296.17 Lm^−2^h^−1^bar^−1^) towards rhodamine B. They inferred that enhanced pollutant removal was due to interactions between the membrane and rhodamine B via hydrogen bonding, pore-filling, coordination effects, electrostatic and π–π interactions.

##### Clay

Clay minerals are extensively applied in adsorption-related applications due to their distinctive surface characteristics. The basal surfaces of most clay minerals possess negative charges across pH 2 to 12. In contrast, the edge surfaces display pH-sensitive charging properties. This difference gives rise to an anisotropic charge distribution within the clay [81]. For instance, vermiculite clay (Ver), composed of octahedral layers of oxygen-aluminum atoms and tetrahedral layers of oxygen-silicon atoms, has a high cation exchange efficiency (100 – >200 meq/100 g). This implies successful exchange of positive ions with surrounding materials [82,83]. Kanmaz et al. [40] explored malachite green adsorption utilizing 10-HKUST-1/Ver. The maximum adsorption capacity from the Langmuir isotherm was 296.74 mg/g for 10-HKUST-1/Ver, and the adsorption mechanism was inferred to be influenced by hydrogen bonding, electrostatic interactions, hydrophobic interactions, and π–π interactions.

Diatomite (DE), on the other hand, primarily consists of silicon dioxide and has cristobalite as one of its dominant mineral phases [84]. Sheng et al. [39] demonstrated that the synthesized Co@MIL-125Ti@DE had superior thermal stability compared with MIL-125Ti. Importantly, Co@MIL-125Ti@DE achieved a maximum adsorption capacity of 622.84 mg/g for sodium fluorescein, exceeding pristine MIL-125Ti (401.55 mg/g).

#### 3.2.3. Natural Biopolymers

##### Cellulose

Microcrystalline cellulose (MCC) units have been introduced in conjunction with MOFs. MCC possesses abundant hydroxyl groups that offer favorable binding sites to trigger hydrogen bonding of pollutants onto MOF composites. The merging of MCC with Ce-BTC (BTC = 1,3,5-benzenetricarboxylic acid) yielded more than double the adsorption observed with either Ce-BTC or MCC alone towards Congo Red (i.e., 81% vs. 38% and 34%) [38]. Furthermore, an adsorption capacity of 926.3 mg/g was achieved at 20 wt% MOF content. The mesoporous nature of the adsorbent enabled the CR moieties to diffuse into the pores and the periphery rapidly. This pore-filling effect caused the pollutant to interact with Ce-BTC@MCC. In addition, CR created hydrogen bonds with the OH groups in MCC CR. The aromatic rings in both the adsorbent and the CR facilitated π–π interactions. In addition, Ce cations at the periphery of Ce-BTC@MCC strongly interacted with N atoms of the amino group in CR through chelation. FTIR and XPS confirmed these interactions.

In addition to CA, other components can simultaneously reduce the *S*_BET_ and enhance pollutant adsorption. This is because their bulky nature reduces the available *S*_BET_. However, the presence of compatible functional groups in these units enhances adsorption. For instance, cellulose acetate (CA) incorporation reduced the surface area of the UiO-66-NH_2_ adsorbent from 529.87 m^2^/g to 28.77 m^2^/g [46]. The composite, UiO@CA, exhibited 91.74% and 88.74% adsorption efficacy for tetracycline and metronidazole, respectively, exceeding those of UiO-66-NH_2_ (79.12%, 76.34%) and CA (49.21%, 41.82%). Such an outcome was linked to the CA providing polar functional groups that supported polar interactions and hydrogen bonding. The presence of the UiO@CA composite membrane enabled size-selective adsorption of the pollutants by allowing water to pass while retaining larger entities (TC and MNZ). The amino groups in UiO-66-NH_2_ were inferred to form hydrogen bonds with the hydroxyl components of MNZ and TC. Also, π–π interactions likely favored TC removal due to the presence of benzene rings. Hydrophobic and Lewis acid–base interactions could play a role during MNZ adsorption. This could account for the higher pollutant adsorption despite the smaller surface area.

##### Chitosan

The introduction of chitosan, another polysaccharide, can provide both amino and hydroxyl groups, which are favorable for organic pollutant adsorption. In an affiliated study, cinnamon bark (CNM) was decorated with cross-linked chitosan (Ch) to form Ch/MIL-101@CNM adsorbents [37]. The corresponding surface area dropped from 27.12 m^2^/g (MIL-101@CNM) to 1.88 m^2^/g (Ch/MIL-101@CNM). A TC removal efficiency of 98.02% was achieved for Ch/MIL-101@CNM (30 mg) after 30 min, which was higher than the 87.43% observed for MIL-101@CNM (60 mg) after 60 min. This demonstrated the pivotal role that chitosan played in the composite despite a lower adsorbent dosage. In detail, they inferred that the hydroxyl and amino groups from the chitosan supplied oxygen and nitrogen atoms to create hydrogen bonds with the polar components in TC. Also, these groups promoted electrostatic interactions between the composite and the pollutant. Then, the terephthalic acid in MIL-101 provided benzene rings that correlated with benzene units in TC, leading to π–π interactions.

##### Cotton

Cotton is an attractive support material for MOF composites owing to its affordability, chemical resistance, biocompatibility, and adaptable surface features [85]. Its active unsaturated surface centers, enriched with carboxyl, amino and hydroxyl functional groups [86], facilitate pollutant interaction. In a related study, UiO-66-NH_2_ was anchored onto cotton fabric using ethylenediaminetetraacetic dianhydride (EDTA) as a cross-linking reagent, yielding EDTA-UiO-66-NH_2_/cotton fabric composites (CFC), which achieved a maximum methylene blue (MB) adsorption capacity of 131.5 mg/g [36]. The governing mechanism involved hydrogen bonding and π–π interaction between the MB benzene ring and the adsorbent, as evidenced by FTIR. Electrostatic attraction existed between cationic MB and the negatively charged composite surface (as witnessed from pH analysis) due to the deprotonation of adsorbent surface functional groups [87].

##### Gum

Xanthan gum (XG) is an anionic polysaccharide formed by the bacterium Xanthomonas campestris through aerobic fermentation [88]. Its primary backbone is formed by a linear chain of β-(1-4)-D-glucose units, a configuration that closely resembles cellulose [89]. This can serve as a biopolymeric backbone owing to its biodegradable and non-toxic nature, along with its hydroxyl- and carboxylate-rich centers, which provide abundant sites for pollutant interaction. Jalil et al. [35] incorporated MIL-101(Fe)/ZnO and 3-aminobenzoic acid (aimed at improving the water insolubility of the adsorbent) onto XG for Rhodamine red adsorption. The plausible mechanism for XG-*g*-P3ABA@MIL-101(Fe)/ZnO was inferred to be hydrogen bonding, π–π stacking, electrostatic and coordination interactions.

In addition, Jabbari et al. [50] developed AG-*g*-PANI@MnFe_2_O_4_/UiO-66-NH_2_ (AG-*g*-PANI = Arabic gum-grafted-polyaniline) for quercetin adsorption. The composite showed 9.1157 mg/g adsorption capacity. The three-dimensional polymer network and mesoporous structure provided accessible adsorption sites. Hydroxyl, carbonyl, and amine groups also promoted hydrogen bonding and electrostatic interactions. π–π interactions further contributed to quercetin uptake. The composite remained effective over five cycles, although efficiency decreased from 73.74% to 65.32%. Similarly, Motallebi et al. [51] developed an AG-*g*-PpPA@MOF(Fe)/Cu (PpPA = poly(p-phenylene diamine) bionanocomposite for cloxacillin adsorption, achieving 400 mg/g. Hydrogen bonding and electrostatic interactions were mainly attributed to amine, carboxyl, and hydroxyl groups. The material retained 78.65% adsorption after 3 cycles.

#### 3.2.4. Soft Polymerics

##### Resin

Synthetic resins, made of quaternary ammonium functionalities on a polystyrene-divinylbenzene backbone, have been widely adopted as adsorption substrates due to their excellent chemical stability and their effectiveness in promoting the accumulation and diffusion of anionic compounds within the resin matrix [90]. MOF immobilization within the resin framework further improves material stability by preventing MOF detachment while preserving the selective interactions of MOFs with target pollutants [91]. In a related study, Huang et al. [34] prepared 2-Zr-La-MOFs@201 to afford 123.6 mg/g adsorption of NTMP (nitrilotris(methylenephosphonic acid)), which was 3.08 and 1.83 times higher than those of the D201 resin and Zr-La-MOFs, respectively. FTIR analysis revealed that hydroxyl groups play a crucial role in NTMP adsorption. Further evidence from XPS indicated that adsorption mainly occurred through strong coordination interactions involving these –OH groups. In contrast, direct Zr–P bonding at coordinatively unsaturated Zr sites was not the dominant adsorption pathway. The abundance of surface –OH groups was largely attributed to defects within the Zr–O clusters and to the presence of La–OH species. Consequently, terminal hydroxyl and water ligands are necessary to compensate for structural defects in the Zr-La-MOF structure [92,93].

DFT calculations were performed to examine the possible adsorption configurations and their associated adsorption energies. NTMP adsorption predominantly occurred via a mononuclear monodentate (MM) M–O–P coordination mode, with a calculated adsorption energy of −4.43 eV, indicating spontaneous adsorption. A stronger interaction was observed when NTMP coordinates directly with unsaturated metal sites to form a bidentate binuclear (BB) inner-sphere complex through M-P bonding, yielding an adsorption energy of −5.61 eV. Additionally, BB coordination can be established through ligand exchange, in which defect-compensating hydroxyl groups are substituted by NTMP, forming M-O-P linkages. Defect-induced exposure of metal active sites not only increases the number of available adsorption centers but also decreases the adsorption energy barrier at existing adsorption sites. The calculated adsorption energies (Eads) of NTMP on UiO-66@201 and La-BDC@201 were −3.73 and −1.24 eV, respectively. Since the magnitude of Eads reflects the affinity between the adsorbent and pollutant, a larger absolute value indicates stronger adsorption interactions. For SO_4_^2−^ adsorption, the calculated Eads values followed the sequence UiO-66@201 (−0.84 eV) < Zr-La-MOFs@201 (−0.71 eV) < La-BDC@201 (−0.08 eV). These findings suggest that the defect sites introduced in Zr-La-MOFs@201 substantially enhance its adsorption affinity toward NTMP. The newly generated active sites facilitate pollutant transport within the framework, ultimately reducing the equilibrium adsorption time.

##### Gel

Gels are a class of three-dimensional polymeric network materials and are typically divided into hydrogels and aerogels. Gelation is initiated when concentrated polymer solutions or sols form a network, leading to a progressive increase in viscosity and ultimately yielding a homogeneous elastic matrix. Benefiting from their facile synthesis, low manufacturing costs, chemical resilience, and versatile functional groups, gel adsorbents have evolved rapidly and are now used in adsorption-based applications [94,95,96]. In an affiliated study, Ye et al. [33] removed 275.8 mg/g of methylene blue after the introduction of CA/PVA@UiO-66 (calcium alginate = CA, polyvinyl alcohol = PVA). This was 135% above the adsorption capacity of pristine CA/PVA. The adsorption behavior was inferred as follows. The planar aromatic structure of methylene blue interacted favorably with the aromatic framework of UiO-66 through π–π interactions. Concurrently, hydroxyl and carboxyl groups on the composite surface served as hydrogen-bond donors and acceptors. Deprotonation of carboxylic groups generated negatively charged sites that enhanced the uptake of cationic methylene blue entities via electrostatic forces. The hierarchical microporous network within the gel spheres provided confinement effects that facilitated capillary condensation and improved adsorption efficiency.

Also, He et al. [48] developed an in-situ grown UiO-66-NO_2_ gel@CMC/SA (carboxymethyl cellulose and sodium alginate) composite aerogel for simultaneous removal of methylene blue and methyl orange. It achieved maximum adsorption capacities of 658.8 and 183.2 mg/g, respectively. The interconnected porous structure of the UiO-66-NO_2_ and the CMC/SA gel improved pollutant accessibility. FTIR and XPS proved that hydroxyl and carboxylate groups contributed through hydrogen bonding and interfacial interactions. Zr sites also interacted with the dyes via metal–ligand coordination. Electrostatic attraction varied with pH, while π–π interactions occurred between the aromatic MOF structure and dye molecules. DFT calculations elucidated the pollutant-adsorbent interaction.

Furthermore, Zhou et al. [49] prepared UiO-66@CPAV (calcium alginate-polymerized vinyl silica-based nanoparticles) by growing UiO-66 within a cross-linked CPAV gel. The composite achieved an MB adsorption capacity of 841.7 mg/g. The adsorbent pores improved MB accessibility and mass transfer. Adsorption involved hydrogen bonding and electrostatic attraction between MB and –OH/–COOH groups. π–π interactions also occurred between the aromatic rings of MB and UiO-66. The combined mesoporous gel and microporous MOF structure further enhanced access to internal sites via capillary adsorption. The composite retained 91.9% of its initial capacity after ten cycles, indicating good structural stability and reusability.

### 3.3. Critical Assessment and Research Gap

#### 3.3.1. Role and Limitations of Metal Sites

Overall, the reviewed studies showed that changing the metal center can influence pollutant adsorption through coordination sites, framework flexibility, pore environment, and metal-adsorbate interactions. Therefore, adsorption performance cannot always be explained by *S*_BET_ alone. However, the specific contribution of the metal center is often difficult to isolate. Metal substitution can also change pore structure, surface area, accessibility, defects, and other textural properties. As a result, it is not always clear whether improved adsorption arises mainly from metal-site chemistry or from changes in accessible porosity. Existing literature similarly emphasizes the combined roles of metal nodes, ligands, pore structure, surface area, and functional groups in determining adsorption behavior.

Future studies should compare different metal centers under closely matched *S*_BET_, pore structures, particle sizes, and activation conditions. Quantitative characterization of adsorption sites should be combined with adsorption experiments and molecular-level modelling. This approach would help distinguish the contribution of metal-site chemistry from that of accessible porosity. It would also provide stronger evidence for understanding the role of metal centers in low-BET MOFs.

#### 3.3.2. Role and Limitations of Auxiliary Components

The reviewed studies demonstrated that auxiliary components, including functional groups, layered materials, polymers, and biopolymers, can modify the adsorption performance of low-BET MOFs. These components may introduce additional binding sites or alter surface chemistry and pore accessibility. However, identifying the main contribution of an auxiliary component can seem difficult. Its incorporation can simultaneously change *S*_BET_, pore structure, surface chemistry, and pollutant accessibility. Therefore, improved adsorption should not be attributed to a single mechanism without sufficient supporting evidence.

Upcoming studies should systematically vary the amount of auxiliary component while keeping other experimental conditions comparable. Adsorption performance should be evaluated alongside *S*_BET_, pore-size distribution, active-site density, and complementary spectroscopic or computational evidence. These comparisons would help determine whether improved adsorption results from additional binding sites, stronger chemical interactions, enhanced pollutant accessibility, or a combination of these effects. This would provide a stronger basis for determining how auxiliary components can offset the effects of limited *S*_BET_ and for guiding the rational design of low-BET MOF adsorbents.

## 4. Adsorbent Synthesis Factors

### 4.1. Synthesis Technique

Various methods have been developed for MOF synthesis, including sonochemical, mechanochemical, hydrothermal, solvothermal, and ionothermal routes. Ionothermal synthesis is gaining popularity because it employs ionic liquids or deep eutectic solvents (DESs) as reaction media and structure-directing agents. With their high solvation ability, negligible vapor pressure, and biodegradability, DESs offer a sustainable alternative for MOF fabrication [97,98]. Choline chloride and sorbitol are widely used in the preparation of DESs because of their low toxicity, biodegradability, cost-effectiveness, and environmental friendliness [99,100,101,102].

From allied research, Mertsoy et al. [32] synthesized copper terephthalate MOFs in DESs (Cu-BDC-D) at 45 °C for 6 h, achieving 98.7% methylene blue removal. These preparation conditions were considerably milder than those required for conventional solvothermal synthesis (120 °C, 24 h), resulting in a 99.2% reduction in energy consumption. In MOF synthesis, DESs serve as effective reaction media by dissolving both organic and inorganic precursors and promoting crystallization through hydrogen-bonding interactions, enabling efficient ionothermal synthesis under relatively mild conditions with reduced energy consumption and shorter reaction times. The adsorption of methylene blue was attributed to electrostatic interactions between the adsorbent and the dye. Furthermore, π–π interactions between the aromatic pollutant rings and the BDC ligands, as well as hydrogen bonding between the amino groups of MB and the carboxylate groups of Cu-BDC, contributed to the adsorption process. These interactions were validated through XPS, FTIR, and pH analyses.

### 4.2. Solvent Type

The use of different solvents during adsorbent synthesis can affect the adsorption capacity and *S*_BET_ of MOFs. In a bid to improve MOF recovery after the adsorption process, cryogels can be employed to favorably maintain the MOF material [103,104]. However, the choice of solvent can result in varying MOF loading into cryogels. For example, ZIF-Co and ZIF-67 were formed when cobalt nitrate and 2-methylimidazolate were dissolved in water and anhydrous methanol, respectively [31]. Boat-shaped sheet crystals and conventional rhombic dodecahedral morphologies were observed from scanning electron microscopy (SEM) images. Upon merging with microfibrillated cellulose (MFC), the resulting MFC/ZIF-Co and MFC/ZIF-67 had 5.79 m^2^/g and 147.2 m^2^/g *S*_BET_, respectively, likely due to larger MFC/ZIF-Co pore sizes (15.47 nm > 3.87 nm) that cause lower nitrogen gas uptake. Despite the smaller value in MFC/ZIF-Co, element distribution spectra revealed that it had higher MOF amounts compared to MFC/ZIF-67. This was reflected in distinctly higher Congo Red (CR) adsorption of 1103.3 mg/g and 813.1 mg/g in MFC/ZIF-Co and MFC/ZIF-67, respectively. The adsorption mechanism relied on hydrogen bonding, electrostatic interactions, and π–π interactions, as indicated by FTIR and XPS analysis.

## 5. Low-Surface-Area MOFs vs. High-Surface-Area Adsorbents

Materials with low *S*_BET_ can still exhibit significant adsorption performance when they possess abundant active sites or strong chemical affinity toward target contaminants. The influence of metal sites, auxiliary components, and synthesis conditions positions them with adsorption potentials that are the same as or even greater than those of larger surface areas. Therefore, low *S*_BET_ materials can remain effective adsorbents despite their limited internal pore structures.

Concerning studies that compared MOFs with identical metal centers/ligands but differing surface areas, MFC/ZIF-Co (5.79 m^2^/g) outperformed MFC/ZIF-67 (147.2 m^2^/g) for Congo Red adsorption [31], as reported earlier. This is a rare direct comparison where lower surface area correlates with higher performance within the same structural family. This shows that, in some cases, a low-surface-area adsorbent can be mechanistically advantageous compared to a high-surface-area adsorbent.

Based on surface area-normalized adsorption, conventional adsorbents (activated carbon, zeolite, and resin) and some benchmark MOFs (MIL-101, ZIF-8, UiO-66) yielded < 1 mg/m^2^ adsorption of pollutants. On the other hand, MIL-101- and ZIF-8-based low-surface-area MOFs yielded adsorption efficacies of 4–>200 mg/m^2^ (Table 2). This indicated highly competitive pollutant adsorption with the low-surface-area MOFs. Because adsorption capacity is normalized by accessible *S*_BET_, higher adsorption densities (mg/m^2^) indicate more efficient utilization of the accessible surface area, suggesting that adsorption is governed by enhanced active-site availability and stronger adsorbate–surface interactions rather than by surface area alone.

**Figure 3:**
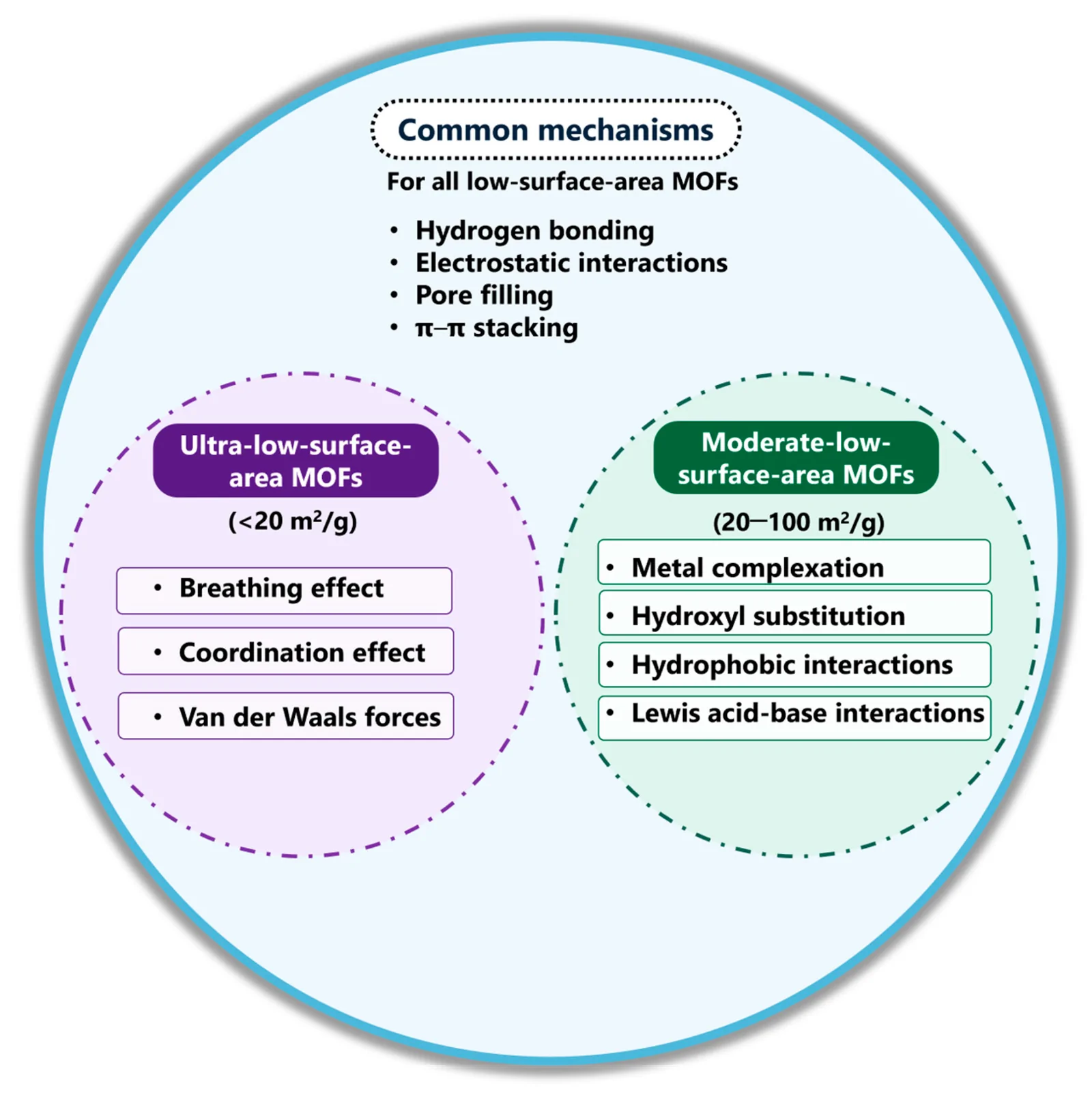
Adsorption mechanisms operating in ultra-low-surface-area MOFs and moderate-low-surface-area MOFs.

All low-surface-area MOFs (BET ≤ 100 m^2^/g) discussed here shared some common mechanistic foundation comprising hydrogen bonding, π–π stacking, electrostatic interactions, and pore filling. However, a clear distinction existed between these two sub-classes (Figure 3): (i) ultra-low-surface-area MOFs (*S*_BET_ < 20 m^2^/g), relied predominantly on the breathing effect, coordination effect, and Van der Waals forces. (ii) Moderate-low-surface-area MOFs (*S*_BET_ = 20–100 m^2^/g) additionally engaged metal complexation, hydroxyl substitution, hydrophobic interactions, and Lewis acid–base interactions. This mechanistic divergence underscores that the transition between these sub-classes reflected not merely a quantitative difference in surface but also a shift in operative adsorption chemistry.

**Figure 4:**
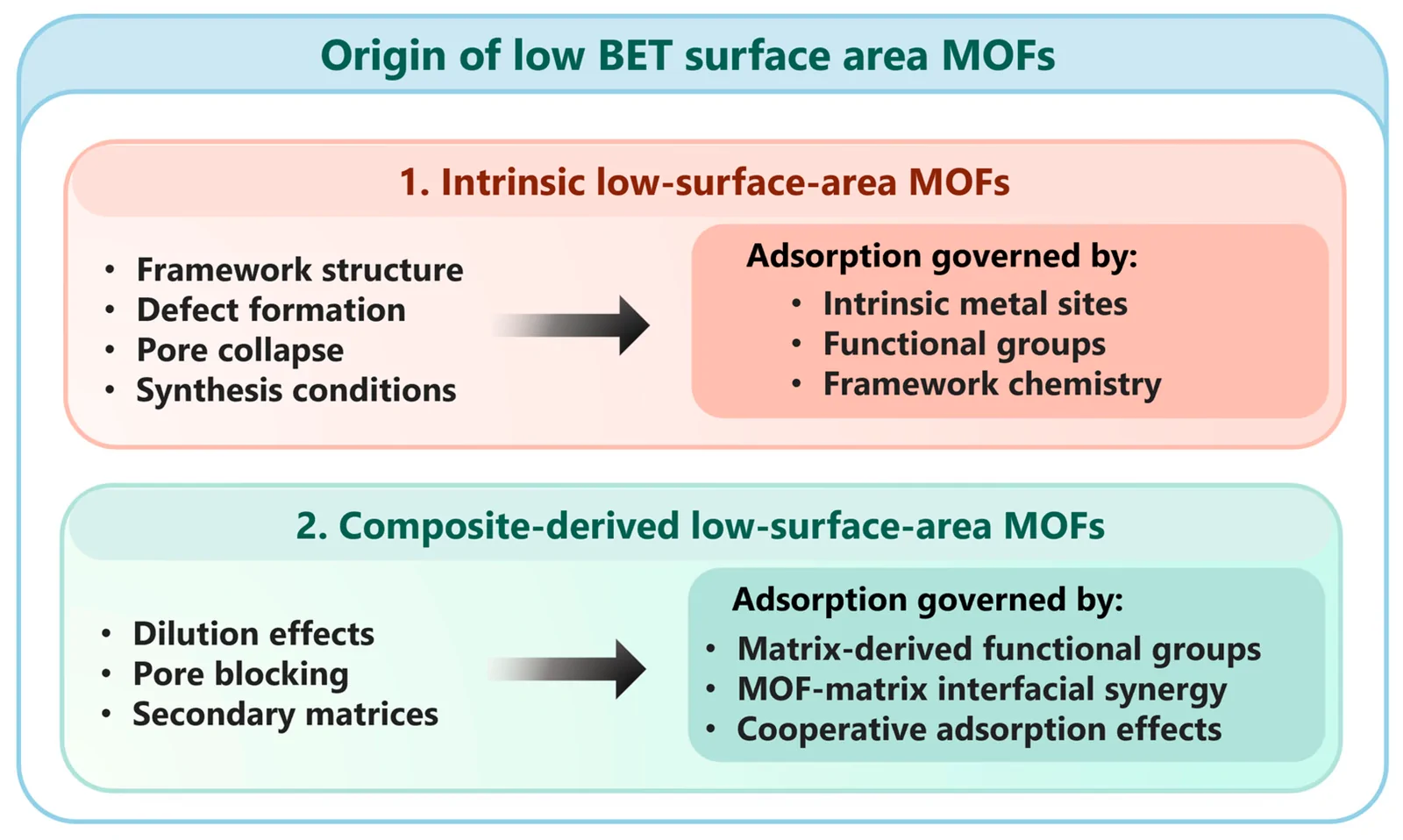
Conceptual classification of the origins of low-BET MOFs reviewed in this study. Low-BET MOF systems are grouped into two broad categories according to the factors responsible for their measured low *S*_BET_: (1) intrinsically low-surface-area MOFs and (2) composite-derived low-surface-area MOFs.

The reviewed materials can be broadly classified into two categories based on the origin of their low *S*_BET_ (Figure 4). The first category consists of composite-derived low-surface-area MOFs, in which relatively high-surface-area MOFs are combined with secondary matrices, leading to dilution effects or pore blocking that reduce the overall *S*_BET_ (Table 3). In such systems, adsorption is predominantly influenced by matrix-derived functional groups, matrix–MOF interfacial synergy, and cooperative adsorption effects. The second category comprises intrinsic low-surface-area MOFs, in which the reduced S_BET_ results from factors such as framework structure, defect formation, pore collapse, or synthesis conditions. In these materials, adsorption is primarily governed by intrinsic metal sites, functional groups, and the inherent framework chemistry (Table 4). The studies showed that adsorption improvements coincided with the introduction of chemically active functional groups despite reductions in S_BET_. For the low-BET MOF systems reviewed here, enhanced adsorption was often associated with favorable surface chemistry and stronger adsorbate–adsorbent interactions, even when the measured *S*_BET_ was relatively low. However, this observation does not imply that porosity is generally secondary to surface chemistry. Accessible surface area, pore size distribution, pore accessibility, framework flexibility, and diffusion can also strongly influence pollutant uptake. Therefore, within the specific low-BET systems examined in this review, surface chemistry can compensate for, and in some cases outweigh, the contribution of measured *S*_BET_, but its relative importance depends on the structural and adsorption characteristics of each material.

### Critical Assessment: Beyond BET Surface Area

The comparative evidence indicated that measured *S*_BET_ alone is insufficient to explain adsorption performance across the reviewed low-BET systems. Several materials exhibit substantially higher adsorption densities than conventional high-surface-area adsorbents, suggesting that accessible functional groups, active-site chemistry, pore accessibility, and adsorbate–surface interactions contribute strongly to pollutant uptake. However, these observations should not be interpreted as evidence that surface chemistry universally outweighs porosity. In many cases, differences in pore size distribution, framework flexibility, active-site density, morphology, pollutant accessibility, and experimental conditions occur simultaneously with changes in *S*_BET_, preventing their individual contributions from being resolved.

A major research gap is therefore the lack of controlled comparisons in which *S*_BET_, pore accessibility, and surface chemistry are independently varied or quantified. Future studies should combine surface-area-normalized adsorption with quantitative active-site measurements and molecular-level characterization to determine whether high adsorption densities arise from greater site density, stronger binding affinity, improved accessibility, or combinations of these factors. Such studies would provide a more rigorous basis for determining when reduced measured surface area can be compensated by favorable surface chemistry and when porosity remains the dominant performance parameter.

These mechanistic uncertainties are accompanied by broader methodological and engineering gaps that limit comparisons among low-BET MOF adsorbents and their practical application in water treatment. As summarized in Table 5, variations in BET measurement and activation procedures can affect the reported surface area. Active-site density and pollutant-accessible porosity are also rarely quantified. In addition, mechanistic interpretations often rely on indirect evidence. The literature is further dominated by single-solute batch experiments, with less attention given to competitive adsorption, real wastewater, regeneration stability, and economic feasibility.

Addressing these gaps requires a more integrated evaluation framework. Future studies should use standardized *S*_BET_ measurements and quantitatively assess active sites and pore accessibility. Complementary spectroscopic, structural, and computational methods should also be used to strengthen mechanistic interpretation. Studies should further examine multicomponent and real-water systems, regeneration stability, and metal or linker leaching. From an engineering perspective, techno-economic analysis (TEA) and life-cycle assessment (LCA) should be incorporated to evaluate production, regeneration, separation, and treatment costs. Fixed-bed and flow-through experiments, followed by pilot-scale validation, are also needed to assess treatment performance. Together, these approaches can improve the reproducibility, mechanistic understanding and practical engineering relevance of low-BET MOF adsorbents.

## 6. Conclusions and Outlook

**Figure 5:**
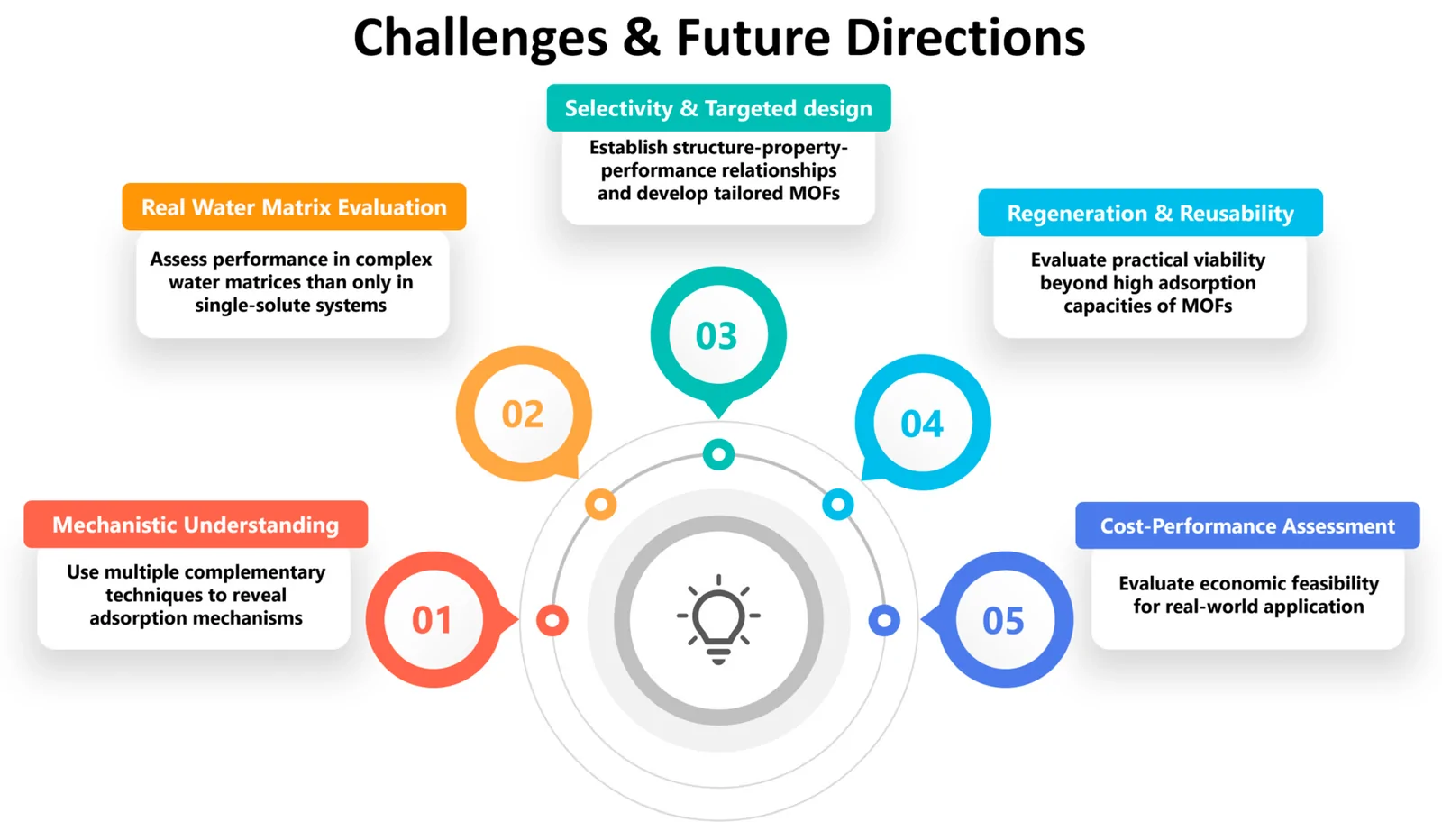
Research priorities for advancing low-BET MOFs from laboratory-scale adsorption studies towards practical water treatment. The framework identifies interconnected priorities including (1) molecular-level validation of adsorption mechanisms using complementary experimental and computational approaches, (2) evaluation under realistic and complex water matrices, (3) quantitative pollutant selectivity and targeted MOF design, (4) regeneration, stability, and metal/ligand leaching assessment, and (5) optimization of synthesis, cost, and scalability.

This review critically examined some low-BET-surface-area MOFs (*S*_BET_ ≤ 100 m^2^/g) as highly effective adsorbents for organic pollutant removal. The compositional modifications involving metal centers, auxiliary components, and synthesis factors were discussed. Cooperative mechanisms that enhance pollutant adsorption capacity were presented. To advance this research further, the following recommendations merit consideration in future investigations (Figure 5):

1. A more rigorous approach is needed to establish the mechanisms responsible for the high adsorption performance of low-BET MOFs. Many mechanisms were inferred from a few characterization techniques, which may provide incomplete evidence. Future work should therefore employ two or more complementary and independent approaches, such as FTIR, XPS, zeta-potential analysis, calorimetric measurements, adsorption modelling, and DFT. Cross-validation would strengthen mechanistic interpretation and distinguish surface chemistry, active-site density, and adsorbate–surface interactions.
2. Water-matrix constituents can influence adsorption through competition for active sites and interaction with dissolved organic matter. Although high adsorption efficiencies have been reported under controlled laboratory conditions, their performance in real wastewater remains insufficiently established. Future studies should evaluate low-BET MOFs in the presence of competing ions, dissolved organic matter, multiple pollutants, and variable pH. Also, mechanisms identified in single-solute systems should be assessed if they remain operative under complex conditions.
3. Limited selectivity toward specific organic pollutants remains an important challenge. Future research should establish relationships between metal–ligand chemistry, functional groups, and pollutant molecular properties. Combining controlled synthesis with DFT and machine-learning approaches could identify descriptors governing pollutant selectivity and facilitate targeted MOF design. Multivariate, mixed-ligand, and multifunctional MOFs should also be explored to create tailored adsorption environments.
4. High adsorption capacity alone does not establish the practical viability of low-BET MOFs. Future studies should emphasize regeneration efficiency, active-site preservation, and potential metal and ligand leaching. Stability under diverse conditions (varying pH, elevated temperatures, or chemically aggressive surroundings) should be evaluated. Robust composite architectures could further improve material recovery and reusability.
5. A comprehensive cost-performance assessment is needed to determine whether the advantages of low-BET MOFs can translate into economically viable treatment technologies. Future evaluations should consider precursor costs, energy consumption, and material lifetime, with emphasis on low-cost precursors and scalable synthesis. Reactor configurations should be optimized while reducing operating costs. Pilot-scale studies using real wastewater are ultimately needed to bridge laboratory findings and practical implementation.

## Figures and Tables

**Table 1:** Structural validation and proposed origins of low *S*_BET_ in the reviewed materials.

Material	Low-Surface-Area Material Category	PXRD Matching	Elemental Composition	Reason for Low *S*_BET_ Value	Ref.
MFC/ZIF-Co cryogel	composite-derived	Matched	XPS, SEM-EDX	Synthesis of ZIF-Co in aqueous solutions, which led to the formation of non-porous structures. Increased pore size reduced N_2_ uptake and *S*_BET_	[31]
Cu-BDC-D	Intrinsic	Matched	SEM-EDX	Small pores of the Cu-BDC hindered the entry of nitrogen molecules	[32]
CA/PVA@UiO-66	composite-derived	Matched	XPS, SEM-EDX	CA/PVA had low surface area (8.2 m^2^/g)	[33]
2-Zr-La-MOFs@201	composite-derived	Matched	XPS, SEM-EDX	201 resin had low surface area (17.7 m^2^/g)	[34]
XG-*g*-P3ABA@MIL-101(Fe)/ZnO.	composite-derived	Matched	SEM-EDX	XG-*g*-P3ABA and ZnO had low surface areas (6.06 and 2.79 m^2^/g)	[35]
EDTA-UiO-66-NH_2_/CFC	composite-derived	Matched	SEM-EDX	CF had low surface area (1.3 m^2^/g)	[36]
Ch/MIL-101@CNM	composite-derived	Matched	SEM-EDX	chitosan caused the internal pore structure to be filled. Also, CNM had low surface area (1.99 m^2^/g)	[37]
Ce-BTC@MCC	composite-derived	Matched	XPS	MCC had low surface area (0.713 m^2^/g)	[38]
Co@MIL-125Ti@DE	composite-derived	Matched	ICP-OES	DE had low surface area (24.28 m^2^/g)	[39]
10HKUST-1/Ver	composite-derived	Matched	SEM-EDX	Ver had low surface area (28.70 m^2^/g)	[40]
CoFe-MOF/GO–TA	composite-derived	Matched	XPS, SEM-EDX	filling of TA and GO in the surface defects of CoFe-MOF	[41]
CBG11	composite-derived	Matched	XPS, SEM-EDX	GO had low surface area (5.3 m^2^/g)	[42]
MIL-88A/NiFe-LDH@SA	composite-derived	Matched	SEM-EDX	SA beads caused structural densification and pore blockage. NiFe-LDH had low surface area (29.77 m^2^/g)	[43]
MIL-88B(Fe)/ZnTi-LDH	composite-derived	Matched	XPS, SEM-EDX	ZnTi-LDH had low surface area (84.241 m^2^/g)	[44]
D-MXene/Co-MOF	composite-derived	Matched	XPS, SEM-EDX	D-MXene of 2.87 m^2^/g	[45]
Cr:Fe(50:50)	intrinsic	Matched	XPS, SEM-EDX	Higher Fe content increased MIL-53 formation, followed by MIL-53 pore closure and N_2_ hinderance	[22]
Fe:In(25:75)-5%-dhba	intrinsic	Matched	XPS	Flexible, non-rigid MIL-88A framework	[23]
UiO-66-NH_2_/cellulose acetate membrane	composite-derived	Matched	Not reported	CA had low surface area (0.5–1.0 m^2^/g)	[46]
1′@MeCl	Secondary ligand	Matched	XPS, SEM-EDX	Methyl functional group partially blocked the micropores	[47]
UiO-66-NO_2_ gel@CMC/SA	composite-derived	Matched	XPS, SEM-EDX	CMC/SA had low surface area (4.84 m^2^/g)	[48]
UiO-66@CPAV	composite-derived	Matched	XPS, SEM-EDX	CPAV had low surface area (53.6 m^2^/g)	[49]
AG-*g*-PANI@MnFe_2_O_4_/UiO66-NH_2_	composite-derived	Matched	SEM-EDX	AG-*g*-PANI@MnFe_2_O_4_ had low surface area	[50]
AG-*g*-PpPA@MOF(Fe)/Cu	composite-derived	Matched	SEM-EDX	AG-*g*-PpPA had low surface area (2.16 m^2^/g)	[51]
PAN@UiO-66 (Zr/Hf)	composite-derived	Matched	SEM-EDX	PAN nanofiber had low surface area	[52]
Zn/Zr-MOFs	intrinsic	Peaks not defined	Not reported	Not reported	[53]
PnBC/ZIF-8-CNs	composite-derived	Weak ZIF-8 peaks	XPS, SEM-EDX	nanocellulose had low surface area (24.47 m^2^/g)	[54]
Ni-MOF and Ni-Fe MOF	intrinsic	No clear peaks for Fe MOF	XPS	Increasing molar ratio of Ni/Fe	[55]
Fe-MOF	Intrinsic	Matched	Not reported	Not reported	[56]
rod-like Tb-MOF	Intrinsic	Matched	XPS, SEM-EDX	Not reported	[57]
Zn-MOF	intrinsic	Matched	SEM-EDX	Not reported	[58]
10CuMMT	composite-derived	Matched	Not reported	Cu-MOF blocked the interlayer spaces of the MMT	[59]
MIL53-NH_2_/XG–Fe_3_O_4_	composite-derived	Matched	Not reported	CPTMS–XG–Fe_3_O_4_ had low surface area (6.43 m^2^/g)	[60]

**Table 2:** Comparison of low-surface area adsorbents with high-surface area adsorbents in terms of surface-area-normalized adsorption of pollutants.

Adsorbents	Qe, mg/g	Surface Area, m^2^/g	Surface-Area-Normalized Adsorption, mg/m^2^	Ref.
High-surface area adsorbents				
MIL-101	420.6	982.19	0.428	[74]
ZIF-8	359.61	1674.17	0.215	[27]
AC	1121.5	2900	0.387	[105]
zeolite	454.55	495.8	0.917	[106]
Resin	214.85	1322	0.163	[107]
Low-surface area MOFs				
AG-*g*-PpPA@MOF(Fe)/Cu	400	4.62	86.580	[51]
Co@MIL-125Ti@DE	622.84	69.08	9.0162	[39]
MIL-88B(Fe)/ZnTi-LDH	543.89	30.97	17.560	[44]
MFC/ZIF-Co cryogel	1103.3	5.79	190.55	[31]
UiO-66-NO_2_ gel@CMC/SA	658.8	19.56	33.689	[48]
UiO-66@CPAV	841.7	96.3	8.740	[49]

**Table 3:** Critical parameters of composite-derived low-BET-surface-area MOFs toward effective adsorption of organic pollutants in water.

MOF Material	Modification	BET Area (m^2^/g)	Total Pore Volume, cm^3^/g	Average Pore Diameter, nm	Pollutant	Qe (mg/g)/Ads. Eff. (%)	Adsorption Mechanism	Reuse Times	Ref.
UiO-66-NH_2_/cellulose acetate membrane	cellulose; acetate membrane	28.766	0.091863	12.774	Metronidazole, Tetracycline, and Rifampin	88.74%, 91.74% and 28.3%	Hydrogen bonding; π–π interactions; polar interactions; hydrophobic interactions; Lewis acid–base interactions; size-selective adsorption.	5 runs,	[46]
UiO-66-NO_2_ gel@CMC/SA	Cellulose; carboxymethyl cellulose and sodium alginate	19.5555	0.0294	5.4238	methylene blue	658.8 mg/g	electrostatic forces, H-bonding, π–π interactions, and metal–ligand coordination	329.5 mg/g after 4 cycles	[48]
20 wt% Ce-BTC@MCC	Cellulose; microcrystalline cellulose (MCC)	67.9	-	104.5	Congo red	926.3	Pore filling; H-bonding; π–π interactions; chelation.	4 runs, 89%	[38]
Ch/MIL-101@CNM	Chitosan	1.88	0.00073	4.96	Tetracycline	33.77	H-bonding; electrostatic interactions; π–π interactions.	5 runs, 85.03%	[37]
Co@MIL-125Ti@DE	Diatomite clay	69.08	0.104	8.299	Fluorescein sodium	622.84 mg/g	Not specified	91.53% after 5 cycles	[39]
10HKUST-1/Ver	Vermiculite clay	50.74	-	-	Malachite green	98.69% 296.74 mg/g	H-bonding; electrostatic interactions; hydrophobic interactions; π–π interactions.	84.34% after 5 cycles	[40]
EDTA-UiO-66-NH_2_/CFC	cotton fabric composite	87.2	-	-	Methylene blue (MB), safranin O (SO), crystal violet (CV)	132.1, 119.1 and 105.1 mg/g	H-bonding; π–π interactions; electrostatic attraction.	MB = 91.3% after 5 cycles	[36]
UiO-66@CPAV	Calcium alginate-acrylic acid- vinyl silica-based gel	96.3	-	17.9	Methylene blue	841.7 mg/g	H-bonding, electrostatic interactions, π–π interactions, and capillary adsorption	91.9% after 10 cycles	[49]
CA/PVA@UiO-66	hydrogel	17.4	-	10.86–15.78	Methylene blue	275.8 mg/g	π–π interactions; H-bonding; electrostatic interactions; confinement/capillary condensation.	259.0 mg/g after 10 cycles	[33]
CuBTC-GO	graphene oxide (GO)	21.8	0.0757	13.22	Tetracycline	39.22 mg/g	H-bonding; electrostatic interactions; π–π interactions; pore penetration/pore filling.	65% after 6 cycles	[42]
CoFe-MOF/GO–TA	graphene oxide; -tannic acid (GO-TA)	6.81	1.5658	9.249	Rhodamine B	>98.1%	H-bonding; pore filling; coordination interactions; electrostatic interactions; π–π interactions.	-	[41]
AG-*g*-PANI@MnFe_2_O_4_/UiO66-NH_2_	Arabic gum -polyaniline (PANI)	9.4971	0.0212	8.9259	Quercetin	9.1157	π–π interactions, electrostatic forces, H-bonding	65.32% after 5 cycles	[50]
AG-*g*-PpPA@MOF(Fe)/Cu	Arabic gum-grafted-poly(para phenylene diamine) (AG-*g*-PpPA)	4.62	0.025	12.51	Cloxacillin	400 mg/g	H-bonds and electrostatic interactions	78.65% after 3 cycles	[51]
XG-*g*-P3ABA@MIL-101(Fe)/ZnO	Xanthan gum (XG) with 3-aminobenzoic acid (3ABA)	10.24	0.05	1.21	Rhodamine B	107.52 mg/g	H-bonding; π–π stacking; electrostatic interactions; coordination interactions.	73.2% after 4 cycles	[35]
Fe:In(25:75)-5%-dhba	Secondary ligands	15.48	0.0371	3.4138	Tetracycline	475.42 mg/g	Pore filling; electrostatic interactions; π–π interactions; H-bonding.	-	[23]
1′@MeCl	Secondary ligands	18	0.013	-	Diclofenac, naproxen, and ibuprofen	482.9, 295.9, and 219.5 mg/g	electrostatic interactions	~ 80% after 3 cycles	[47]
PAN@UiO-66 (Zr/Hf)	Bimetallic sites	99.18	-	-	Naproxen	164.20 mg/g	Metal complexation, coordination, H-bonding, π–π interactions, and electrostatic interactions	82.8% after 5 cycles	[52]
MIL-88B(Fe)/ZnTi-LDH	LDHs	30.9733	0.04643	10.6020	Tetracycline	543.89 mg/g	Electrostatic interactions.	119.57 mg/g after 3 cycles	[44]
MIL-88 A/NiFe-LDH@SA	Layered double hydroxides (LDHs)	85.89	0.131	7.17	Tartrazine	94.82%	H-bonding; π–π interactions; electrostatic interactions.	85% after five cycles	[43]
MFC/ZIF-Co cryogel	Solvent type	5.79	0.022	15.47	Congo red	1103.3 mg/g	H-bonding; electrostatic interactions; π–π interactions.	4 runs, 70%	[31]
2-Zr-La-MOFs@201	D201 resin	60.1	0.417	3.2641	Nitrilotris(methylenephosphonic acid)	123.6 mg/g	M–O–P coordination; inner-sphere complexation; electrostatic interactions; defect-site-assisted adsorption.	>90% after 5 cycles	[34]

**Table 4:** Critical parameters of intrinsic low-BET-surface-area MOFs toward effective adsorption of organic pollutants in water.

MOF Material	Modification	BET Area (m^2^/g)	Total Pore Volume, cm^3^/g	Average Pore Diameter, nm	Pollutant	Qe	Adsorption Mechanism	Reuse Times	Ref.
D-C2-M	MXene	21.35	0.036	1.29	Tetracycline	47.66 mg/g	Nanochannel diffusion/pore accessibility, π–π stacking, electrostatic interactions, and H-bonding.	10 runs, >95%	[45]
Cr:Fe(50:50)	Metal type	8.9	0.015	3.980	P-Nitroaniline	378.9 mg/g	Flexible-framework/pore-opening mechanism, π–π interactions, and H-bonding.	69.71% after 5 cycles	[22]
Cu-BDC-D	Synthesis method	60.3	0.052	2.13	Methylene blue	195.19 mg/g	H-bonding, π–π interactions, and electrostatic interactions	80.92% after five cycles	[32]

**Table 5:** Evidence gaps, limitations and research priorities for low-BET MOF adsorbents.

Research Dimension	Current Evidence	Main Limitation	Information Still Needed	Recommended Approach	Ref.
BET surface area	Widely reported as a primary textural descriptor	BET values can vary with activation history and measurement conditions	Reproducible surface-area values and complete reporting of activation/measurement conditions	Standardized activation protocol; report adsorption isotherms, and fitting range	[30,108]
Active-site density	Active sites are rarely quantified	Site abundance and accessibility are often not quantitatively determined	Site density and accessibility	Combine quantitative site characterization with spectroscopy and structural analysis	[109]
Adsorption mechanism	FTIR, XPS, zeta potential and adsorption modelling are frequently used	Individual techniques may not uniquely distinguish mechanisms	Molecular-level identification and independent validation	Combine FTIR/XPS with structural analysis and DFT calculations	[110]
Pore accessibility	N_2_-BET surface area and pore-size distributions are commonly reported	Gas-accessible surface area does not necessarily represent pollutant-accessible surface area in aqueous systems	Accessibility of pores (volume and entrances) to the actual aqueous pollutant, rather than only N_2_-accessible surface area	Combine N_2_ adsorption with pore-size analysis, water-phase adsorption measurements and molecular simulations	[108]
Selectivity	Many studies use single-solute adsorption experiments	Single-solute tests do not adequately represent competitive adsorption	Selectivity under multi-solute and realistic water conditions	Binary/multicomponent adsorption, competing ions and real-water experiments	[111]
Regeneration	Reusability tests are reported in a subset of studies	Short cycling tests may not establish long-term structural or chemical stability	Capacity retention, structural integrity, active-site preservation and metal/linker leaching	At least >5 cycles as a practical screening target, with PXRD/FTIR and ICP-MS/ICP-OES before/after cycling	[112]
Real wastewater	A limited number of studies evaluate real wastewater or complex matrices	Synthetic single-solute water remains common; matrix effects are insufficiently characterized	Effects of DOM, competing ions, salinity, pH and other matrix constituents	Test real wastewater, conduct controlled DOM and competing-ion experiments. Include realistic pollutant concentrations.	[113]
Cost	Material performance is frequently reported without complete economic analysis	Laboratory-scale adsorption capacity does not directly establish economic feasibility	Adsorbent production cost, regeneration cost, energy demand and cost per volume treated	Techno-economic analysis (TEA) and life-cycle assessment (LCA), including synthesis, regeneration, separation, transport, and disposal	[114]
Scale-up	Batch adsorption experiments dominate the literature	Comparatively few continuous-flow, fixed-bed or pilot-scale demonstrations	Mass-transfer parameters, pressure drop, regeneration and long-term operation	Fixed-bed/flow-through experiments followed by pilot-scale validation	[115,116]

## Data Availability

Data will be available on request.

## Supplementary Materials

The following supporting information can be downloaded at https://www.mdpi.com/article/10.3390/nano16191271/s1: Table S1: Summary of the chemical attributes, major uses, and health effects of organic contaminants adsorbed using MOFs with smaller BET surface areas.
