# Supplementary material for: Challenging the Surface Area Paradigm: A Critical Review of Low-BET Metal–Organic Frameworks for the Adsorption of Organic Water Pollutants
Source: Nanomaterials (Basel). 2026 Oct 8;16(19):1271. doi: 10.3390/nano16191271 (PMC13667223; doi:10.3390/nano16191271)
Supplement: Supplementary file 1 [file nanomaterials-16-01271-s001.zip › nanomaterials-4587388-supplementary.pdf]

Supplementary Information

Challenging the Surface Area Paradigm: A Critical Review of Low-BET Metal-Organic Frameworks for the Adsorption of Organic Water Pollutants

Malcom Frimpong Dapaah [1], Xingbao Zhu [2,*], Hui Jia [3], Huiying Zhang [4], Ruth Antwi-Baah [5], Candy Ann Dwamena Darkwa [6] and Liang Cheng [3,*]

[1] School of Agricultural Engineering, Jiangsu University, Zhenjiang, 212013, China; mfdapaah@ujs.edu.cn
[2] Technical Centre for Soil, Agriculture and Rural Ecology and Environment, Ministry of Ecology and Environment, Beijing 100012, China; zhuxingbao@tcare-mee.cn
[3] School of the Environment and Safety Engineering & Institute of Environment and Ecology, Jiangsu University, Zhenjiang 212013, China; 1000005403@ujs.edu.cn (H.J.); clcheng@ujs.edu.cn (L.C.)
[4] School of Environment, Tsinghua University, Beijing, 100084, China; ying_zz1995@163.com
[5] College of Ecology and Environment, Chengdu University of Technology, Chengdu, 610059, China; maadede97@gmail.com
[6] Department of Materials and Metallurgical Engineering, College of Engineering, Kwame Nkrumah University of Science and Technology, Kumasi, AK-448-7038, Ghana; candydarkwa@gmail.com
* Correspondence: zhuxingbao@tcare-mee.cn (X.Z.); clcheng@ujs.edu.cn (L.C.)

Table S1. Summary of the chemical attributes, major uses, and health effects of organic contaminants adsorbed using MOFs with smaller BET surface areas

Organic pollutant	Chemical structure	Molecular weight (g/mol)	Major uses	Health concerns	Refs.
Acid Fuchsin ($C_{20}H_{17}N_3Na_2O_9S_3$)	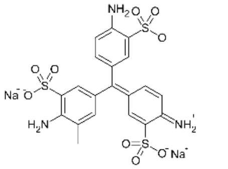	585.54	Dye, disinfectant	Carcinogenic, respiratory system	[26,27]
Aniline blue ($C_{37}H_{29}N_3O_9S_3$)	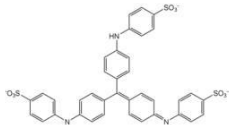	755.8	Staining animal tissues, industrial dye	Affects the gastrointestinal tract, kidney and liver, causes bladder cancer	[28]
Ciprofloxacin ($C_{17}H_{18}FN_3O_3$)	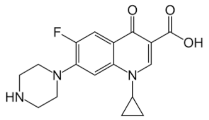	331.35	antibiotics	Extended diarrhea	[29,30]
Cloxacillin ($C_{19}H_{18}ClN_3O_5S$)	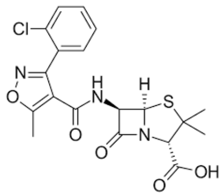	435.88	Treat skin infections and respiratory tract infection	Antibiotic resistance, environmental toxicity	[31,32]
Congo red ($C_{32}H_{22}N_6Na_2O_6S_2$)	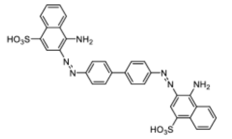	696.664	dyes in culinary, cosmetics, textiles, and medicinal industries	Carcinogenic	[33]
Crystal Violet ($C_{25}N_3H_{30}Cl$)	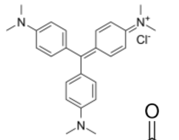	408	Industrial dye	Chemical cystitis	[34,35]
Diclofenac Sodium ($C_{14}H_{10}Cl_2NNaO_2$)	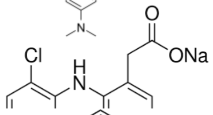	318.13	Anti-inflammatory drugs	Gastrointestinal concerns	[36,37]
Fluorescein Sodium ($C_{20}H_{10}Na_2O_5$)	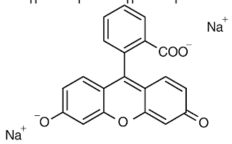	376.27	Organic dye	Kidney damage, allergies	[38]
Ibuprofen ($C_{13}H_{18}O_2$)	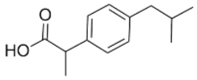	206.285	non-steroidal anti-	Embryotoxicity, cytogenotoxicity, inhibition of	[39,40]

			inflammatory drug	reproduction, and mortality in fish	
Levofloxacin HCl (C18H21ClFN3O4)	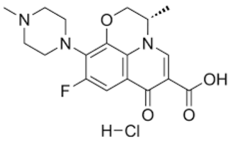	397.831	Antibacterial agent	Emergence of resistance genes	[41]
Lomefloxacin HCl ($C_{17}H_{19}F_2N_3O_3$)	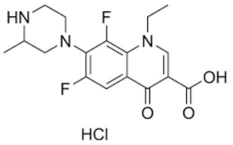	351.354	Antibiotic in veterinary treatments	Gastrointestinal bleeding, liver abnormality	[42]
Malachite Green ($C_{23}H_{25}ClN_2$)	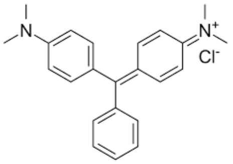	364.911	Industrial dye	Adversely affects the reproductive and immune systems	[43]
Methylene Blue ($C_{16}H_{18}ClN_3S$)	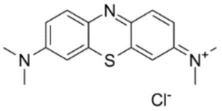	319.85	Industrial dye	Cardio-related issues	[44]
Metronidazole ($C_6H_9N_3O_3$)	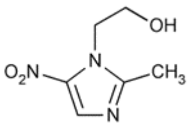	171.154	antibiotic in clinical and veterinary settings	Neurotoxicity, convulsions, and peripheral neuropathy	[45]
Naproxen ($C_{14}H_{14}O_3$)	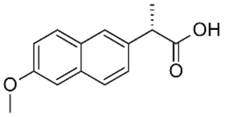	230.26	nonsteroidal anti-inflammatory drug	endocrine disruption and heart disease	[46]
Neutral Red ($C_{15}H_{17}ClN_4$)	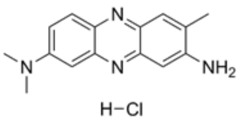	288.77	As dyes in manufacturing process of inks, textiles, leathers, foods and pharmaceuticals	Carcinogenic	[47]
Nitrilotris(Methylenephosphonic Acid) ($C_3H_{12}NO_9P_3$)	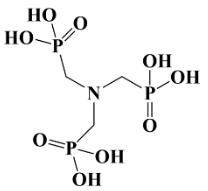	299.05	Chelating agent	Can lead to eutrophication	[48]

Orange G	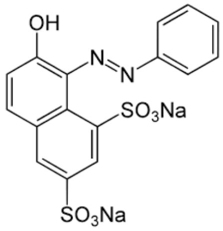		Dye in leather and textile industries	Affects the respiratory and gastrointestinal tract	[49]
Paranitroaniline ($C_6H_6N_2O_2$)	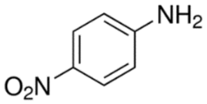	138.12	intermediate for pharmaceuticals, pesticides, dyes, antioxidants, plastics	Damage to the spleen, kidney and liver	[22,50]
Quercetin ($C_{15}H_{10}O_7$)	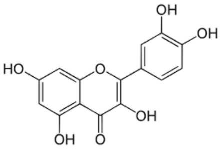	303.23	Immune system regulation	Can be carcinogenic	[51,52]
Rhodamine B ($C_{28}H_{31}ClN_2O_3$)	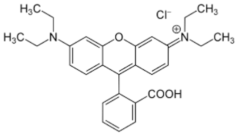	479.02	Dye industries	Nasal discomfort, itchy skin	[53,54]
Rifampin ($C_{43}H_{58}N_4O_{12}$)	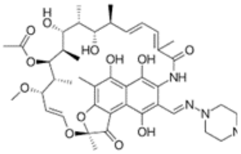	822.9	Antibiotic that acts against mycobacterial species.	Chronic toxicity and endocrine disruption	[55]
Safranin O ($C_{20}H_{19}ClN_4$)	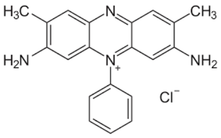	350.85	For biological labs and textile industries	Affects the respiratory tract and damages the cornea	[56]
Tartrazine ($C_{16}H_9N_4Na_3O_9S$)	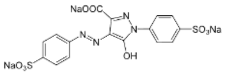	534.36	Dye in cosmetics and pharmaceuticals	Eczema, asthma attacks	[57]
Tetracycline Hydrochloride ($C_{22}H_{24}N_2O_8.HCl$)	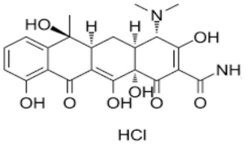	480.90	Antibiotic drugs	kidney and liver issues	[58,59]
